# Measuring Exposure to Gun Violence and Risky Behavior: Psychometric Validation and Analysis of the Gun Violence Exposure (Gun-X) Scale

**DOI:** 10.1007/s11121-026-01916-0

**Published:** 2026-05-01

**Authors:** Kimberly J. Mitchell, Victoria Banyard, Patrick J. Guziewicz, Bruce G. Taylor

**Affiliations:** 1 Crimes Against Children Research Center, University of New Hampshire, 10 West Edge Drive, Suite 106, Durham, NH 03824 USA; 2 School of Social Work, Rutgers University, New Brunswick, NJ USA; 3 NORC at the University of Chicago, Chicago, IL USA

**Keywords:** Gun violence exposure, Psychometrics, Bystander, Scale development

## Abstract

Gun violence is a critical public health problem in the United States, requiring improved strategies for early identification and prevention. This study introduces and validates the Gun Violence Exposure (Gun-X) Scale, designed to assess awareness of gun violence, threats, and risky firearm behaviors within social networks, including in-person and digital contexts. Data were drawn from a nationally representative sample of 5,311 youth and young adults (ages 10–34) and collected from September 2023 to January 2024. Using a multi-method psychometric approach, findings supported a unidimensional structure with good reliability and consistent model fit across training and validation samples. Item Response Theory analyses indicated high discrimination across items and strongest measurement precision at moderate levels of exposure. Convergent validity was supported through associations with violence exposure, peer gun carrying, and neighborhood risk, while discriminant validity was demonstrated with mental health and social support measures. The 10-item Gun-X Scale provides a reliable and generalizable measure of bystander exposure to gun violence. It has applications in research, clinical screening, and prevention efforts, particularly for characterizing exposure patterns and informing tailored, context-sensitive responses. The scale is intended to assess exposure and should not be used as a standalone tool for selecting individuals for intervention roles.

## Introduction

Gun violence continues to be a significant public health concern in the United States. In 2023, 46,728 people died from gun-related injuries (Gramlich, 2025; Kim et al., 2025). Over half (58%) of these deaths were suicide and 38% were murders. Furthermore, 79% of the murders in the United States involve a firearm as do 55% of suicides. The long-standing problem of mass shootings, 355 since 2013 (Gun Violence Archive, 2021), have renewed the public’s interest in preventing firearm violence. Theories of crime prevention often discuss the importance of informal social control – of communities building collective efficacy and giving all members a role to play in violence prevention (Sampson et al., 1997). Bystanders are a key element of informal social control. They are third parties who witness violence, who may know about students carrying firearms to school, or who may know about someone’s plan to use a firearm for violence as expressed on social media. Violence prevention initiatives are increasingly involving these third parties in prevention training, seeing them as potentially important gatekeepers in prevention efforts. There is already evidence that bystander action can reduce intimate partner violence, sexual assault, stalking, and bullying in young adults and adolescents (Banyard, 2008; Banyard et al., 2007, 2014; Edwards et al., 2015; Espelage et al., 2012; Frye, 2007; Hamby et al., 2016; McCauley et al., 2013; Potter, 2012; Warner, 2014). And, most recently, there is increasing attention to bystander action as a possible youth violence prevention strategy (Weisburd et al., 2014). We know very little about this role as a possible prevention tool for firearm exposures. Tools to assess exposure and bystander opportunities would be useful to gather data to use in prevention programs (informing primary prevention by producing information that can be shared to engage community members and increase their awareness of bystander roles), showing community members, for example, the frequency and types of bystander exposures people experience.

Given the success of bystander-focused prevention tools for reducing other risk behaviors, it seems that a logical next step is to explore the roles that bystanders might play in relation to reducing risky firearm use (Staub, 2019). For example, people could be trained to tell others, including responsible adults or authorities, about inappropriate firearm access or intent to use. Research on other forms of violence shows that there are unique barriers and facilitators by type of violence (Bennett et al., 2014; Weitzman et al., 2020). In addition, experiences as a bystander may serve as either a risk or protective factor for an individual’s own future firearm violence involvement, either victimization or perpetration. Improved assessments can expand practitioners’ ability to identify these lived experiences which may affect how community audiences engage with bystander-focused gun violence prevention programs. In this paper, we distinguish between three related but conceptually distinct constructs: (1) *bystander exposure*, defined as awareness of gun violence, threats, or risky firearm behaviors within one’s social network; (2) *bystander role potential*, referring to opportunities to intervene or seek help; and (3) *bystander intervention behavior*, referring to actions taken to prevent harm. The Gun-X Scale is designed specifically to assess the first construct - bystander exposure, not intervention behavior or readiness.

To date, measures of what we are describing here as “exposure” have either been rather narrow (e.g., witnessing use of guns) or broad (e.g., community violence). Research on witnessing gun violence often asks about seeing someone get shot (Bancalari et al., 2022; Comer & Connolly, 2023; Leibbrand et al., 2021), seeing someone attacked or threatened with a gun (Bancalari et al., 2022), and hearing gun shots in a public place (Mitchell et al., 2021). These are important contexts for bystander action, though they are arguably the most risky and dangerous situations and may not be the most effective main focus for bystander-focused prevention. This existing research does not capture bystander exposures to threats of violence, through social media or other communications, that suggest escalating risk. Another body of work, on community violence, often uses a broad array of measures, some of which focus on firearm use, but which often include other types of crime exposure that go beyond a focus on bystanders to risky firearm use (DeCou & Lynch, 2017; Kennedy & Ceballo, 2014; Stein et al., 2003). Measurement tools that can screen for bystander opportunities across a range of risky situations where bystanders might be helpful in the prevention of escalation and harm are needed. They could be used in a number of ways: to collect data on the prevalence of bystander opportunity that can be presented in prevention programs to help participants anticipate their own roles as helpers, to help communities identify and discuss potential bystander roles, as well as opportunities and resources needed to support them. Stronger assessment tools can help enhance gun violence prevention programs.

This study addresses the lack of standardized tools for assessing bystander exposure to gun violence by developing and validating the Gun Violence Exposure (Gun-X) Scale. By capturing a wide range of exposures, including direct, indirect, and digital, the scale equips researchers to better study the strengths and challenges of engaging bystanders in gun violence prevention. It would give practitioners a tool that could be used within prevention programs to help participants identify past bystander opportunities (increasing self-awareness and prevention engagement) and to understand the range of experiences of participants to better connect prevention materials to lived experiences of participants.

### Current Study

This study aims to address the gap in standardized scales that assess exposure to gun violence, risky behavior, and inappropriate access or usage. By validating this scale, this study will offer a more complete understanding of the distinct ways people know about gun violence in their social networks, including gun violence, threats, and risky or inappropriate access. It will provide researchers, clinicians, and communities with a screening tool that comprehensively captures multiple types of exposures. This can be a first step in prevention strategies to mobilize bystanders safely and to enable clinicians to start prevention conversations with patients. Importantly, the Gun-X Scale is intended as a measure of exposure and awareness rather than a standalone tool for identifying individuals appropriate for intervention roles. Its primary purpose is to characterize the range and distribution of exposure experiences that may inform prevention, clinical conversations, and future research.

## Methods

### Participants

Data were collected from 5,311 youth and young adults who were part of the AmeriSpeak panel, a nationally representative panel of US households. With over 55,000 US residents, the AmeriSpeak panel is designed to be representative of the US household population using probability-based sampling. Panel members are recruited using address-based probability sampling that covers approximately 97% of US households, with sample weights provided to support population inference (Montgomery et al., 2018). Details of recruitment into the AmeriSpeak panel are published elsewhere (Montgomery et al., 2018). From the panel households, individual residents who were eligible to participate in the study were youth or young adults, ages 10–34 years old, who could speak or read either English or Spanish. Selecting this age range enabled the analysis of a broad developmental span in exposure patterns. Demographic details of the weighted sample are presented in Table 1.

**Table 1:** Sample demographic characteristics

**Characteristic**	**All participants** ***n* (%)**
All participants	*N* = 5311
Age category	
10–17	1189 (30.2)
18–24	853 (28.2)
25–34	269 (41.6)
Mean age	25.07 (SD = 7.0)
Birth sex	
Female	3282 (49.6)
Male	1908 (47.9)
Intersex	17 (0.3)
Missing	104 (2.3)
Gender identity^a^	
Cisgender female	3174 (47.4)
Cisgender male	1870 (47.1)
Gender minority	166 (3.6)
Missing	101 (1.9)
Sexual minority identity (any)	1047 (18.3)
Race	
White	2803 (62.9)
Black or African American	946 (14.2)
Asian	535 (7.1)
American Indian or Alaska Native	63 (0.8)
Native Hawaiian	20 (0.4)
Other race	281 (4.1)
Two or more races	523 (7.5)
Prefer not to answerMissing	140 (3.0)
Hispanic or Latino ethnicity	1104 (22.4)
Not Hispanic or Latino	4149 (77.6)
Annual household income	
Less than $30,000	1273 (22.8)
$30,000 to under $60,000	1373 (24.2)
$60,000 to under $100,000	1260 (24.2)
$100,000 or more	1365 (28.0)
Missing	40 (0.9)
Type of community	
Urban	2260 (36.9)
Suburban	2311 (48.8)
Rural	740 (14.3)
High neighborhood disorder	1530 (27.0)
Poor home conditions	1204 (21.9)

Weighted percentages, unweighted *N*

### Procedures

Randomly selected AmeriSpeak panelists were sent a description of the study by email, and an invitation to complete the survey. Incentives of $20 were provided for those participants who completed the survey. The survey completion rate among those sampled for this study was 33.0%. The survey was administered from September 2023 to January 2024. 

NORC at the University of Chicago’s Institutional Review Board approved the project for data collection. The research team obtained voluntary and informed consent from all participants either by the participant consenting verbally for those completing a survey by phone or by clicking a time-stamped box for those completing the online version of the survey. Participants under the age of 18 provided assent after caregiver consent was obtained. Cognitive testing of the survey instrument (*n* = 5 with some youth under 18 years old and some over age 18), including questions about firearm exposures, helped ensure wording was appropriate for the age range of participants.

### Measures

#### Exposure to Firearm Violence, Threats, or Risky Firearm Behavior (Gun Violence Exposure (Gun-X) Scale)

We developed a series of 10 questions for the current study that queried exposures to someone else’s firearm violence, threats, and risky behaviors. Items were drawn from prior work about youth gun violence exposure which included focus groups and cognitive interviews with youth as young as age 10 (Beseler et al., 2020). These were designed to expand upon previous measures that tend to focus mainly on seeing or being present when someone was shot (Lanfear et al., 2023) or census tract numbers of firearm homicides (Martin et al., 2022). Before these questions, participants were told that we were only asking about things they may have seen or heard about in real life, not things they may have seen on TV, in a movie, on the news or in a video game. The questions asked, “Have you ever…” (yes/no): (1) seen someone shooting a gun in a public place (such as on the street, or a parking lot, school, or store); (2) heard (but not seen) a gun being shot in a public place (such as the streets, parking lots or stores); (3) heard or seen anyone you know talking or posting about hurting someone else with a gun; (4) seen someone or heard anyone threaten to hurt someone else with a gun; (5) heard someone of any age talk about getting a gun or having a gun when they are not supposed to; (6) known someone of any age who had a gun when or where they were not supposed to; (7) known about anyone bringing a gun to school or work? Do not include situations where someone carried a gun to work because that was part of their job, like police or security officers; (8) been surprised or worried because someone you know was carrying a gun (not for their job); (9) heard or seen anyone you know talking or posting about hurting themselves with a gun; and (10) known anyone who has killed or tried to kill themselves with a gun?

#### Convergent Validity

Convergent validity is determined by examining relationships between variables that are theoretically linked to the latent construct that the Gun-X Scale is intended to measure: exposure to and awareness of gun violence, threats, risky behaviors, and inappropriate access. If there is evidence of acceptable convergent validity, it supports the conclusion that the scale is measuring the construct of interest. A total of 19 variables were used in the evaluation. Four variables were dichotomous, asking respondents to indicate (1) whether or not they could get a gun if they wanted to, (2) whether they had ever carried a gun for purposes other than for hunting, target shooting, or a gun safety class, (3) whether they had ever witnessed a shooting in their neighborhood that resulted in injury or death, and (4) whether they had ever witnessed assault without a weapon. Using these items to assess convergent validity is rooted in established empirical relationships. Peer group gun carrying is associated with individual gun carrying (O’Connor et al., 2023) and exposure to the gun violence is also associated with individual gun carrying (Comer & Connolly, 2023). Similarly, higher exposure to community violence is related to individual gun carrying (Yang et al., 2025). Gun access has also been tied to exposure to gun violence by way of individual gun carrying (Chavez et al., 2022).

Respondents were asked questions regarding the frequency with which they experienced both financial and food insecurity on a five-point ordinal scale ranging from “never” to “always.” Food insecurity was assessed by asking, “In the past 30 days, how often did you skip meals or eat less because you or your family didn’t have enough money for food?” Financial insecurity was assessed by asking, “In the past 12 months, how often did your family not have enough money to pay the bills?” Respondents were also presented with a list of 12 neighborhood problems and asked to indicate whether the problem was “no problem,” “a minor problem,” or “a serious problem” in their neighborhood (“by neighborhood, we mean the street you live on and a few streets around it”). Problems queried were gangs, graffiti, drugs, homelessness, damaged buildings, abandoned/boarded-up buildings, violent crime, sirens at night, gun shots at night, fighting, litter or trash, and break-ins or burglaries.

Neighborhood and household disadvantage variables are theoretically linked to bystander gun violence exposure based on established empirical relationships. Specifically, research has documented that neighborhood disadvantage is associated with greater community exposure to deadly gun violence among youth (Kravitz-Wirtz et al., 2022) and with higher rates of gun violence in more socially vulnerable neighborhoods, which tend to show concentrated incident patterns (McMillan et al., 2024), thereby creating more opportunities for bystander exposure. Similarly, household disadvantage indicators such as food insecurity have been associated with increased risk of exposure to multiple forms of interpersonal violence. Thus, if the Gun-X Scale validly captures bystander exposure to gun violence, it should correlate positively with these environmental risk indicators. The inclusion of these variables alongside direct violence exposure measures (witnessing assault, witnessing shootings, peer gun carrying) provides a more comprehensive test of convergent validity across both proximal (direct violence exposure) and distal (environmental context) indicators.

#### Discriminant Validity

Discriminant validity is the extent to which the scale discriminates between the construct it is intended to measure and theoretically unrelated concepts. A total of 12 items were used to assess discriminant validity of the Gun-X Scale, representing two conceptual categories. Five mental health indicators were used from the Mental Health Inventory (MHI-5) (Ware & Sherbourne, 1992). Response options were on a six-point ordinal scale from “none of the time” to “all of the time.” Respondents were asked to indicate how frequently they had experienced five different indicators of mental wellbeing and distress in the past 30 days. These indicators were (1) “been a very nervous person?”; (2) “felt calm and peaceful?”; (3) “felt downhearted and sad?”; (4) “been a happy person?”; and (5) “felt so down in the dumps that nothing could cheer you up?” Items were coded such that higher scores indicated worse mental health problems.

The second conceptual category used in our assessment of discriminant validity was perceived social support. To assess this, respondents were asked to indicate the degree to which they felt each of a series of seven statements was true about social support they received when they were last upset about something. Responses were on a four-point ordinal scale ranging from “not true” to “mostly true.” The statements were (1) “Someone was there for me when I was having a heard time”; (2) “Someone gave me a place where I could get away for a while”; (3) “Someone helped me get my mind off things”; (4) “Someone went with me to get some help”; (5) “Someone comforted me”; (6) “Someone stood up for me when I was in a tough spot”; and (7) “Someone inspired me to work hard.” Responses were coded such that higher scores indicated more perceived social support.

Both the mental health and social support variables were included in the analysis of discriminant validity as they are theoretically distinct from the latent construct measured by the Gun-X Scale. Exposure to firearm violence, threats, or risky behaviors is but one type of many possible antecedents to differences in mental health between individuals. Because of this, there may be some relationship between the two variables, but that relationship is likely to be either weak or non-existent in our large sample given the likelihood that mental health is frequently altered by things other than exposure to gun violence. As such, mental health is a good candidate for testing discriminant validity of the Gun-X Scale.

In the case of social support, there is some evidence in the criminological literature that social support may serve as a buffer against criminal and risky behavior (Colvin et al., 2002). However, while there are some connections between social support and both experiencing and perpetrating *direct* violence (Azimi & Daigle, 2020), there is little empirical basis for a relationship between social support and exposure to someone else’s firearm violence, threats, or risky behavior. Social support can be robust across different kinds of peer groups, including those with both high and low rates of delinquency (Brezina & Azimi, 2018). In contrast, exposure to weapon-involved violence varies between social networks (Tracy et al., 2016). In other words, there is evidence that social support and indirect gun victimization operate independently from one another, making it a strong candidate for establishing discriminant validity.

#### Demographic Characteristics

Demographic characteristics measured include age, birth sex, gender identity, sexual identity, race, and ethnicity. Details of categories within each are reported in Table 1.

### Data Analysis

The analyses were conducted on 5,311 participants who completed the Gun Violence Exposure (Gun-X) Scale. The 10-item Gun-X Scale underwent a comprehensive multi-method psychometric evaluation designed to establish its dimensionality, measurement precision, reliability, and validity. The analytical approach integrated network psychometrics, item response theory (IRT), classical test theory (CTT), and confirmatory factor analysis (CFA) to provide converging evidence for the scale’s properties.

The process began with exploratory graph analysis (EGA; EGAnet::EGA) to assess the scale's dimensionality and identify its underlying factor structure, using a Gaussian graphical model with EBIC-glasso regularization for binary data. EGA simultaneously estimated the dimensionality and derived the factor structure through community detection algorithms. Following initial structure identification, we evaluated structural stability through 5,000 parametric bootstrap resamples, calculating the proportion of times each item was consistently assigned to the same factor. Item redundancy was assessed by examining the weighted partial-correlation network for items with multiple high-weight edges (|*r*|> 0.30 in ≥ 3 connections). In network psychometric analysis, item redundancy is assessed by examining the weighted partial-correlation network, in which edges represent the unique associations between items after controlling for all other items in the network. Specifically, an item was flagged as potentially redundant if it exhibited three or more partial correlations exceeding |*r*|> 0.30 with other items. This threshold indicates that an item shares substantial unique variance with multiple other items, suggesting it may be capturing overlapping content that is already measured by other items in the scale. The rationale is that in an optimally efficient scale, each item should contribute unique information; an item with multiple strong partial correlations may be “over-connected,” indicating it largely duplicates information captured elsewhere. In our analysis, no items met this redundancy criterion; that is, no item participated in three or more partial correlations exceeding |0.30|. This finding, combined with the high edge density (0.756) observed in the network, indicates that while items are strongly interconnected (as expected for a coherent unidimensional construct), each item nonetheless contributes unique information about bystander exposure to gun violence. The items share conceptual space without being redundant.

Following the network analysis, we conducted an exploratory factor analysis to examine item loadings and potential cross-loadings to confirm the factor structure, using tetrachoric correlations, which are appropriate for binary data. Factor extraction used minres with oblimin rotation. Items were evaluated based on factor loadings (criterion ≥ 0.40) and absence of problematic cross-loadings (≥ 0.30). Items failing these criteria were flagged for potential removal. IRT analysis was conducted using a 2-parameter logistic (2PL) model (mirt::mirt) for evaluating item characteristics. This enabled examination of item discrimination (a parameters) and difficulty (b parameters), providing insight into how well items differentiate between levels of exposure, with discrimination values categorized as low (≤ 0.65), moderate (0.65–1.35), high (1.35–1.70), or very high (> 1.70). Model fit was evaluated using the outfit and infit statistics.

CTT evaluation utilized tetrachoric correlations to assess internal consistency, employing Cronbach’s alpha and item-total correlations (criterion ≥ 0.30). For cross-validation, we employed a 30/70 train-test split, using confirmatory factor analysis with weighted least squares mean and variance adjusted (WLSMV) estimation, which is appropriate for binary data. Convergent and discriminant validity were assessed using Multi-Trait Multi-Method (MTMM) analysis, examining correlations between the GWG Bystander Exposure Scale and theoretically related constructs (e.g., neighborhood disorder, witnessing violence, gun access) versus unrelated constructs (e.g., current mental health symptoms, social support). We also included an additional analysis to examine age as a correlate of gun violence bystander exposure.

## Results

### Exploratory Graph Analysis

**Fig. 1:**
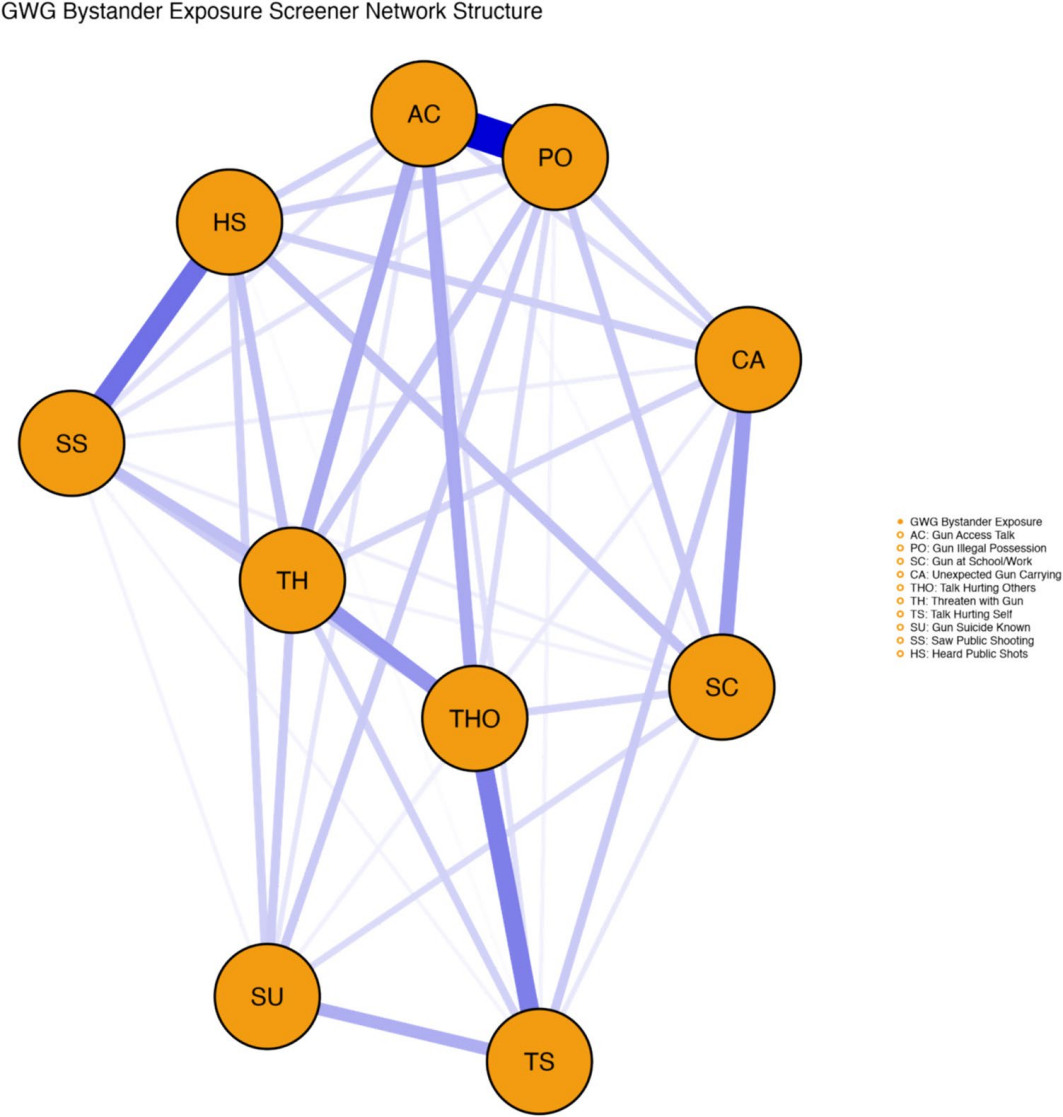
Exploratory graph analysis (EGA) of the 10-item Gun-X Scale reveals a robust unidimensional structure. Each node represents a screening item, with thicker edges indicating stronger regularized partial correlations among items. The single identified dimension encompasses diverse forms of gun violence exposure: AC (gun access talk), PO (gun illegal possession), SC (Gun at School/Work), CA (unexpected gun carrying), TH (talk hurting others and threaten with gun; note: two TH nodes represent related threat items), TS (talk hurting self), SU (gun suicide known), SS (saw public shooting), and HS (heard public shots)

The initial EGA on 10 items reveals a unidimensional structure (see Fig. 1). The GLASSO network (EBIC *γ* = 0.5, *λ* = 0.080) produced 34 edges among 10 nodes with high edge density (0.756), indicating strong interconnections among all items, supporting the idea that they all measure the same underlying concept. The Louvain community detection algorithm identified a single community, confirming that all items measure a unified construct of bystander exposure to gun violence.

Bootstrap stability analysis (5,000 resamples) demonstrated perfect robustness with a median of 1 dimension (SE = 0.00; 95% CI [1, 1]). Remarkably, 100% of bootstraps replicated the single-factor solution. All items achieved perfect stability coefficients (1.000), indicating exceptional measurement stability across bootstrap samples. This shows that the scale’s structure is highly stable and reliable; every time the analysis was repeated, the same result was found. No items met the redundancy criterion of participating in three or more partial correlations exceeding |0.30|, confirming that each item contributes unique information to the scale despite the high interconnectedness.

### Exploratory Factor Analysis

Factor analysis confirms the unidimensional structure identified by EGA (Table 2). The single factor explained 47% of the total variance, indicating good model coverage for a unidimensional construct that measures a specific phenomenon. All items demonstrated strong primary loadings, ranging from 0.546 to 0.807, which is well above the 0.40 criterion, suggesting that each item contributes meaningfully to the overall scale. The highest loading items were “heard someone talk about getting a gun” (0.807) and “known someone who had a gun illegally” (0.792), while items with moderate loadings included “known about gun suicide” (0.546) and “gun brought to school/work” (0.548). No items demonstrated weak primary loadings (< 0.40) or problematic cross-loadings (≥ 0.30), as the unidimensional structure precluded cross-loading issues. Consequently, all 10 items were retained for the final scale.

**Table 2:** Psychometric properties of the Gun-X Scale

Description	*n* (Yes)	% Yes	Discrimination (a)	Difficulty (b)	Factor Loading	Item- Total r
Heard talk about getting a gun	1514	28.5	2.837	0.674	0.807	0.570
Known someone with illegal gun	1816	34.2	2.624	0.497	0.792	0.562
Gun brought to school/work	1212	22.8	1.124	1.343	0.548	0.367
Surprised/worried by gun carrying	1027	19.3	1.274	1.445	0.588	0.387
Talk/post about hurting others	705	13.3	2.173	1.427	0.761	0.473
Witnessed gun threat	1181	22.2	2.023	1.008	0.760	0.515
Talk/post about self-harm	548	10.3	1.654	1.835	0.664	0.389
Known gun suicide/attempt	1298	24.4	1.104	1.262	0.546	0.362
Saw public shooting	771	14.5	1.490	1.617	0.643	0.401
Heard gunshots in public	2575	48.5	1.446	0.064	0.670	0.445

*N* = 5311. Discrimination categories: low (≤ 0.65), moderate (0.65–1.35), high (1.35–1.70), very high (> 1.70). Higher difficulty values indicate items endorsed at higher levels of the latent trait

### Item Response Theory Analysis

**Fig. 2:**
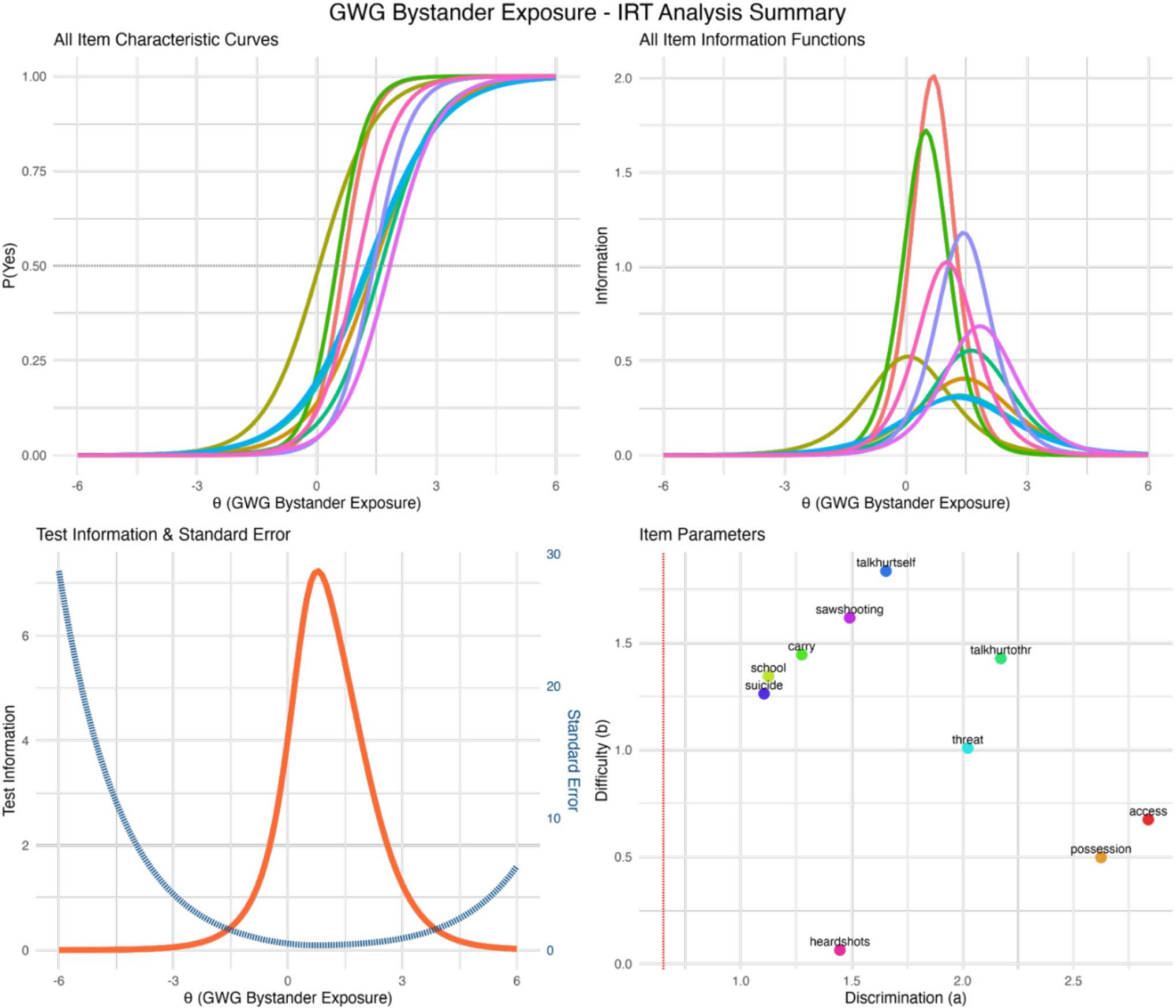
Item response theory (IRT) analysis summary for the Gun-X Scale. **Top left:** item characteristic curves showing the probability of endorsement (P(“Yes”)) as a function of the latent bystander exposure level (θ) for all 10 items. Steeper curves indicate higher discrimination, with items shifting from left to right based on difficulty. **Top right:** item information functions displaying the measurement precision each item provides across the latent trait continuum. Items with higher peaks contribute more information, with heard talk about getting a gun (access) and knowing someone with an illegal gun (possession) providing maximum information around θ = 1. **Bottom left:** test information function (orange) and standard error curve (blue-dotted) demonstrating that the screener achieves peak measurement precision (information = 7.5) at moderate exposure levels (θ−1), with acceptable precision (SE < 1) maintained across the range of − 2 to + 4. **Bottom right:** item parameters plot showing discrimination **a** on the x-axis and difficulty **b** on the y-axis. The vertical red line indicates the minimum acceptable discrimination threshold (*a* = 0.65), which all items exceed. Items in the upper right quadrant, such as talk/post about self-harm (talkhurtself) and talk/post about hurting others (talkhurtothr), require both high discrimination and higher levels of exposure for endorsement, while items in the lower right quadrant, such as heard gunshots in public (heardshots) and known someone with an illegal gun (possession), are highly discriminating but endorsed at lower exposure levels

The two-parameter logistic (2PL) IRT model provides detailed insights into item functioning along the latent bystander exposure continuum (see Fig. 2). Discrimination parameters ranged from 1.104 to 2.837, with 5 of 10 items (50%) exceeding *a* = 1.70 (“very high” discrimination). The most discriminating items were “gun access talk” (*a* = 2.837) and “illegal possession known” (*a* = 2.624). Difficulty parameters spanned 0.064 to *b* = 1.835. Lower difficulty values were observed for “heard gunshots” (*b* = 0.064). In contrast, items such as “talk of self-harm with gun” (*b* = 1.835) and “saw public shooting” (*b* = 1.617) require higher latent exposure levels before endorsement.

The test information function peaked around θ = 1.0, with maximum information approximately 7.5, meaning the scale is most precise for people with moderate levels of exposure, but still provides useful information across a wide range. The corresponding Standard Error curve reached its minimum (< 0.4) near the same θ value, indicating the scale provides the greatest precision for detecting moderate to moderately high levels of bystander exposure. Information remained above 2.0 for θ values between approximately − 1.0 and + 3.0, confirming adequate precision across the clinical range. Item fit statistics indicated excellent model fit, with all items showing acceptable outfit (range 0.464–0.857) and infit (range 0.802–0.993) values. No items required removal based on IRT criteria, supporting the psychometric integrity of all 10 items.

### Classical Test Theory and Reliability

The 10-item scale demonstrated good internal consistency, with Cronbach’s *α* = 0.778 and McDonald’s omega total = 0.810, exceeding the recommended threshold of 0.80 for screening instruments using the omega coefficient. The omega hierarchical value of 0.650 indicates that 65% of the variance in total scores can be attributed to the general factor, while the omega total of 0.810 represents the proportion of total score variance due to all sources of common variance. The discrepancy between the omega total and the omega hierarchical (0.810–0.650 = 0.160) suggests some multidimensionality, though the general factor remains dominant.

Item-total correlations ranged from 0.362 to 0.570, with all items exceeding the 0.30 criterion. The strongest item-total correlations were observed for “gun access talk” (0.570) and “illegal possession known” (0.562), consistent with their high discrimination in IRT analysis. No items would improve alpha if deleted, with all deletions resulting in decreased reliability (alpha reductions ranging from − 0.008 to − 0.037).

### Confirmatory Factor Analysis: Training and Validation

**Fig. 3:**
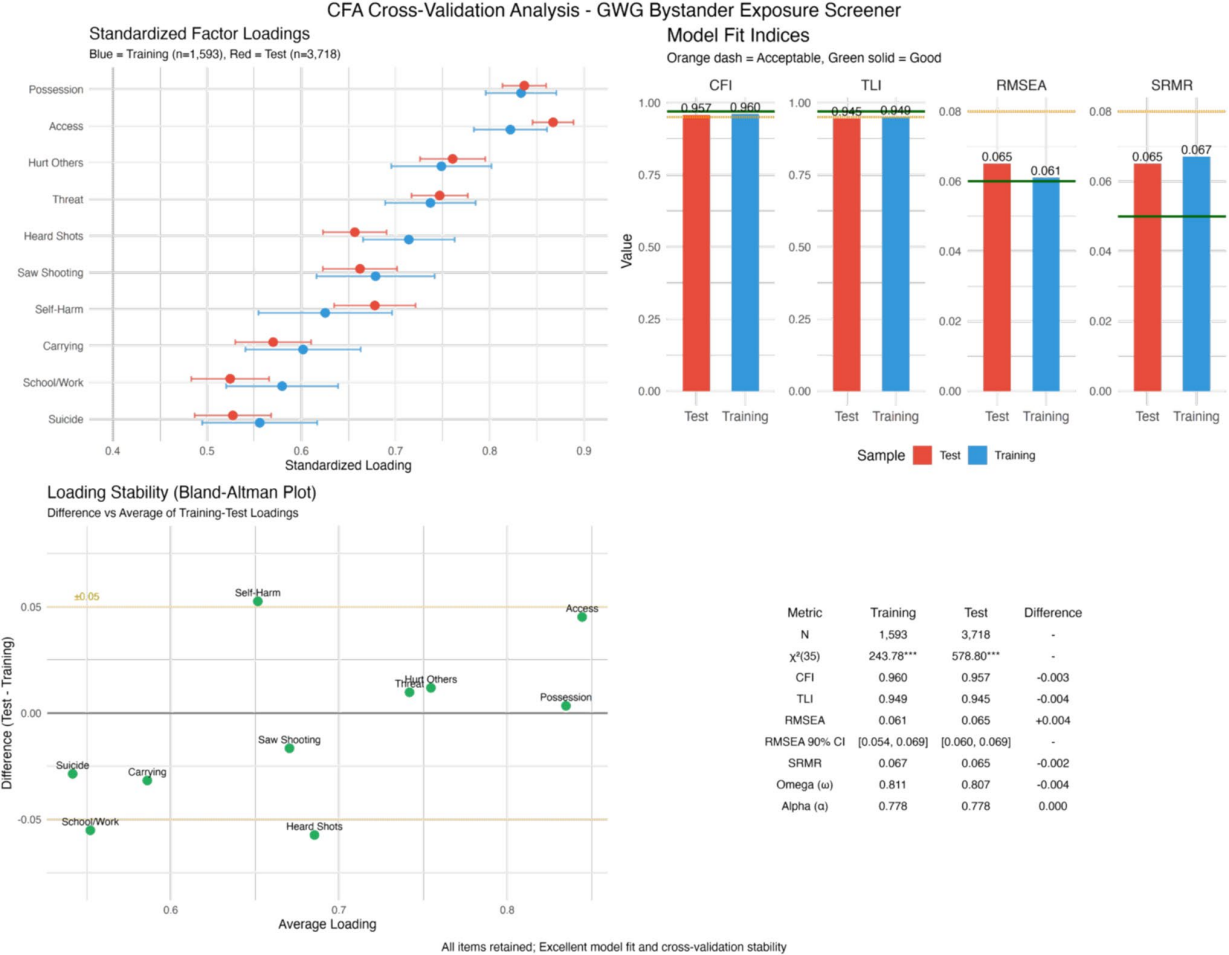
Confirmatory factor analysis (CFA) cross-validation results for the Gun-X Scale. **Top left:** standardized factor loadings with 95% confidence intervals comparing training (*n* = 1593; blue) and test (*n* = 3718; red) samples. All items exceed the minimum loading threshold of 0.40, with “known someone with illegal gun” (possession) and “heard talk about getting a gun” (access) showing the strongest loadings (> 0.80) and excellent consistency across samples. **Top right:** model fit indices for both samples demonstrating excellent fit, with all indices exceeding acceptable thresholds (red dashed lines) and approaching excellent criteria (green dotted lines). CFI and TLI values > 0.95, RMSEA values < 0.07, and SRMR values < 0.08 confirm robust model fit. **Bottom left:** Bland–Altman plot assessing loading stability, where the difference between test and training loadings is plotted against their average. All items fall within the ± 0.05 band (orange dashed lines), with “talk/post about self-harm” (self-harm) showing the largest but still acceptable difference, indicating exceptional measurement invariance. **Bottom right:** summary statistics table showing minimal differences between samples across all metrics (ΔCFI = − 0.003, ΔTLI = − 0.004, ΔRMSEA = + 0.004, ΔSRMR = − 0.002), with reliability coefficients (omega) exceeding 0.80 in both samples. The consistency of results across independent samples supports the generalizability and structural validity of the unidimensional measurement model

To evaluate model stability and generalizability, we conducted a confirmatory factor analysis (see Fig. 3). By “generalizability” in this context, we refer specifically to cross-validation stability, the degree to which the scale's factor structure and model fit replicate in an independent hold-out sample not used for model estimation. We employed a 30/70 train-test split design: the single-factor CFA model was estimated on the training set (*n* = 1593) and then validated on a completely independent test set (*n* = 3,718). The near-identical fit indices across samples (ΔCFI = − 0.003, ΔTLI = − 0.004, ΔRMSEA = 0.001, ΔSRMR = − 0.002) provide evidence that the measurement model generalizes beyond the specific sample on which it was developed. This internal cross-validation approach addresses concerns about model overfitting and demonstrates that the scale's psychometric properties are robust rather than sample-specific.

The precision findings from IRT (test information function, standard error curves) complement this by showing the trait levels at which the scale provides most accurate measurement. Together, these analyses address both structural generalizability (consistent factor structure) and measurement precision (where along the latent continuum the scale performs best).

#### Training Set (*n* = 1593)

The single-factor model demonstrated excellent fit: *χ*^2^ (35) = 243.779, *p* < 0.001; CFI = 0.960; TLI = 0.949; RMSEA = 0.061 (90% CI [0.054, 0.069]); SRMR = 0.067. Standardized factor loadings ranged from 0.556 to 0.833, with all items loading significantly (*p* < 0.001). Composite reliability (omega) was 0.811, exceeding the 0.80 threshold for good reliability.

#### Test Set (*n* = 3718)

The model replicated well in the larger test sample: *χ*^2^(35) = 578.802, *p* < 0.001; CFI = 0.957; TLI = 0.945; RMSEA = 0.065 (90% CI [0.060, 0.069]); SRMR = 0.065. Standardized factor loadings ranged from 0.525 to 0.867, maintaining the same pattern as the training set. Composite reliability (omega) was 0.807, confirming consistent reliability across samples. Model comparison revealed minimal differences between training and test sets (ΔCFI = − 0.003, ΔTLI = − 0.004, ΔRMSEA = 0.001, ΔSRMR = − 0.002).

### Discriminant and Convergent Validities

**Fig. 4:**
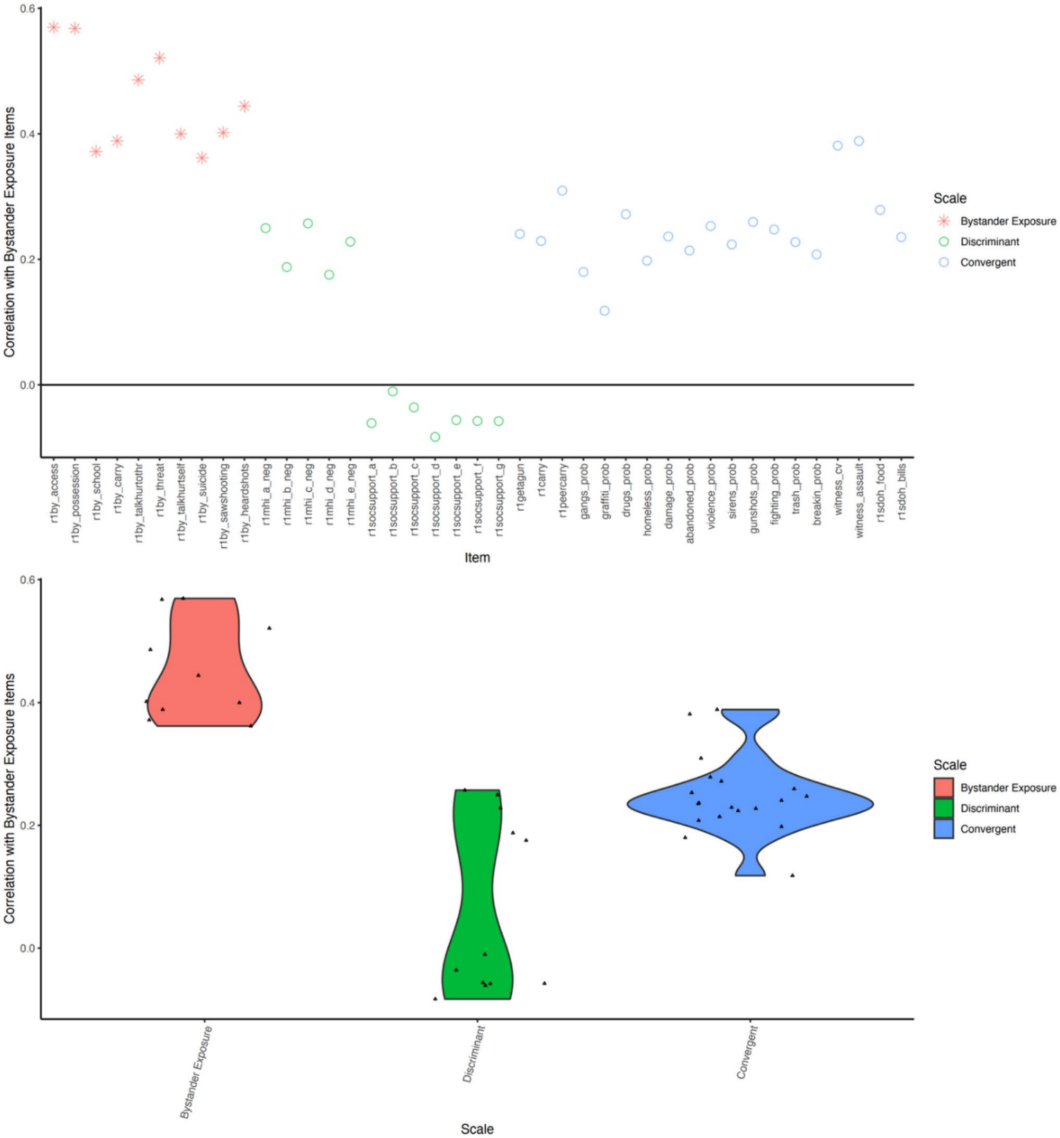
Multitrait-multimethod (MTMM) analysis demonstrating convergent and discriminant validity of the Gun-X Scale. **Top panel:** Item-level correlations between each screener item and external variables. Red stars indicate correlations among bystander-exposure items: heard talk about getting a gun, known someone with an illegal gun, gun brought to school/work, surprised/worried by gun carrying, talk/post about hurting others, witnessed gun threat, talk/post about self-harm, known gun suicide/attempt, saw public shooting, and heard gunshots in public. Green circles represent convergent validity items, including direct gun access and carrying (e.g., could get a gun, ever carried a gun, peer gun carrying), and neighborhood disorder indicators (e.g., gangs, drugs, violence, gunshots, fighting). Blue circles represent discriminant validity items, including mental health symptoms and social support measures. **Bottom panel:** violin plots showing the distribution of correlations by validity type, with convergent correlations substantially higher than discriminant correlations, supporting the scale’s construct validity

MTMM analysis demonstrates strong validity evidence (see Fig. 4). convergent validity was supported by meaningful correlations with theoretically related constructs (mean *r* = 0.247, range 0.118–0.389). The highest correlations were with witnessing violence indicators: witnessed assault (0.389), witnessed shooting with injury/death (0.381), and peer gun carrying (0.309). Strong associations were also observed with neighborhood disorder items, particularly violence problems (0.254), gunshots heard in the neighborhood (0.260), and drug problems (0.272).

Discriminant validity was demonstrated by substantially lower correlations with theoretically unrelated constructs (mean *r* = 0.061, range − 0.083 to 0.257). Mental health symptoms showed weak associations (*r* = 0.175–0.257), while social support items showed near-zero or slightly negative correlations (*r* = − 0.083 to − 0.010). There was a clear distinction between convergent correlations (mean = 0.247) and discriminant correlations (mean = 0.061), with convergent correlations approximately four times larger. Notably, mental health items showed slightly higher correlations than expected (up to *r* = 0.257). However, these correlations remain substantially lower than those with direct violence exposure measures, supporting the discriminant validity of the scale.

### Age as a Correlate of Gun Violence Exposure

Age demonstrated a significant positive correlation with total Gun-X scores (*r* = 0.177, *p* < 0.001; Spearman ρ = 0.180). This relationship is best understood as reflecting cumulative exposure opportunity: older individuals have simply had more time and life experiences during which bystander exposure could occur. ANOVA comparing the three developmental groups revealed significant differences, *F*(2, 5308) = 129.2, *p* < 0.001, *η*^2^ = 0.05. Post-hoc comparisons showed that adolescents (10–17 years; *M* = 1.43, SD = 1.97) reported significantly lower exposure than both young adults (18–24 years; *M* = 2.77, SD = 2.45) and adults (25–34 years; *M* = 2.63, SD = 2.41), while the two older groups did not differ significantly from each other.

This pattern, with exposure increasing from adolescence to young adulthood but plateauing thereafter, is consistent with the interpretation that the age-exposure relationship reflects cumulative lifetime opportunity rather than developmental differences in exposure risk per se. The finding that 52.6% of adolescents reported any exposure compared to approximately 77–78% of older groups supports this cumulative exposure interpretation.

Item-level analyses revealed that the age correlation was strongest for items reflecting more severe or less frequently occurring exposures (e.g., known someone who attempted/completed suicide by gun, *r* = 0.188; known someone with illegal gun possession, *r* = 0.139), while items capturing more immediate social network behaviors (e.g., hearing someone talk about hurting others, *r* = 0.019, ns) showed minimal age associations. This differential pattern further supports the cumulative opportunity interpretation: rarer events accumulate more noticeably over time. 

## Discussion

Gun violence continues to be a significant public health concern, and we need to find new and innovative ways to approach prevention, specifically those that help stop gun violence before it occurs. Using an approach that has growing popularity in other violence prevention efforts, both interpersonal (bullying, sexual assault) (Banyard, 2015) and self-directed (suicide) (Banyard et al., 2024; Mitchell et al., 2022), we examine how third parties know about potential gun violence by those in their social networks. A strong assessment measure has utility across the spectrum of prevention strategies. At the universal, or primary prevention level, a robust and broad assessment of potential bystander opportunities enables researchers to better estimate the prevalence of various bystander opportunities, a foundation for raising awareness in communities and potentially engaging community members in prevention training. At the secondary level, the Gun-X Scale can help identify individuals and groups with higher levels of exposure who may benefit from further assessment and tailored prevention approaches, rather than serving as a standalone indicator for bystander intervention training. Finally, given what we know about how gun violence exposure can negatively impact well-being and mental health, a broad assessment scale can be part of tertiary or indicated assessments to fully capture the adversity burden that individuals or groups may be coping with to start conversations to promote coping and resilience. To this end, the current study aimed to develop and validate the Gun-X Scale to assess the range of different ways people are exposed to gun violence, threats and risky behavior.

The Gun-X Scale captures experiences that may constitute forms of vicarious or indirect trauma, as individuals are exposed to threats, violence, and risk within their social networks. This has important implications for prevention programming. Individuals with high levels of exposure may carry an emotional burden or stress related to these experiences, and bystander-intervention efforts should incorporate trauma-informed approaches, including attention to emotional safety, coping resources, and appropriate boundaries around intervention expectations.

Importantly, the Gun-X Scale should not be used as a standalone selection tool for bystander intervention training. While higher scores indicate greater exposure and potential opportunity for intervention, they may also reflect proximity to high-risk environments or involvement in gun-related behaviors. As such, we recommend that Gun-X Scale be used within a multi-stage screening framework that incorporates additional assessment of behavioral history, current risk, and readiness to intervene. For example, individuals reporting prior involvement in gun violence or unsafe firearm behaviors may be more appropriately referred to targeted prevention or support services rather than bystander training programs.

Findings indicate that the Gun-X Scale has excellent psychometric properties, including structural stability and robust evidence of convergent and discriminant validity. Results of the exploratory graph analysis indicate that gun violence bystander exposure represents a coherent, integrated construct rather than multiple distinct domains. The absence of problematic items supports the coherence and specificity of the construct being measured.

The IRT findings indicate the most discriminating items were “gun access talk” and “illegal possession known,” indicating these items sharply distinguish between individuals at different exposure levels. Lower difficulty for “heard gunshots” indicates this experience is endorsed even at relatively low overall exposure levels, likely reflecting the ambient nature of gunshots in certain communities. Items such as “talk of self-harm with gun” and “saw public shooting” required higher latent exposure levels before endorsement probability exceeded 50%, suggesting these represent more severe exposure experiences.

The CFA findings further strengthened the validity of the Gun-X Scale by confirming its cohesive structure. The excellent model fit indices suggest that, while gun violence exposure can manifest in various forms, such as hearing gun shots in public places, risky gun carrying, threats of interpersonal violence, and someone else’s social thoughts and behaviors involving guns, these behaviors are interconnected. Indeed, the 10-item scale demonstrated good internal consistency, with Cronbach’s *α* = 0.778, indicating the items reliably measure a coherent construct despite capturing diverse exposure experiences. The model comparison between training and test sets found minimal differences, demonstrating exceptional cross-validation stability. The consistent factor loadings and fit indices across independent samples support the robustness and generalizability of the unidimensional measurement model.

MTMM analysis demonstrated strong validity evidence. Specifically, convergent validity was indicated given the strong correlations with witnessing assault, witnessing shooting with injury/death, peer gun carrying, and violence problems in the neighborhood, hearing gunshots and drug problems. This confirms that the Gun-X Scale captures exposure to gun violence within broader community violence contexts. Discriminant validity was also seen with low correlations with constructs that are not theoretically expected to be related to gun violence exposure (mental health and social support). This provides strong evidence that the scale specifically measures gun violence exposure rather than general psychological distress or social functioning. Notably, mental health item correlations were slightly larger than expected suggesting that bystander exposure to gun violence may have modest associations with psychological symptoms. This is supported by literature that documents the impact of indirect forms of gun violence (Lennon et al., 2023; Mitchell et al., 2021; Turner et al., 2019).

### Limitations

While the Gun-X Scale demonstrates strong psychometric properties, there are some limitations that warrant consideration. While the scale provides a valuable tool for assessing the variety of ways people know about gun violence, risky behavior, and inappropriate access or usage, it does not capture the full complexity of these situations. Nuances of the situation are critical for understanding the risk potential to all parties involved. The scale may be overly inclusive, capturing experiences that might not ultimately be classified as actual or possible gun violence. It is important for any bystander-focused prevention training, but particularly with gun violence, to discuss the limitations of accurate identification of gun risk as well as potential safety concerns if individuals or groups try to do something.

The Gun-X Scale is a next step in improving research on bystander roles, and in generating data about bystander opportunities that can be the basis for nuanced conversations in communities about bystanders and violence prevention. Although higher Gun-X scores may be associated with environments or social networks in which risk for gun violence is elevated, the scale was not designed as a diagnostic or predictive tool for perpetration. Any use of the scale to identify individuals at elevated risk for committing violence would require additional validation and should be approached cautiously to avoid misclassification or stigmatization.

Reliance on self-reported data introduces potential recall bias, and future research could benefit from qualitative or mixed-methods approaches to better understand the nuances of gun violence exposure. As such, findings should be interpreted with caution, especially when considering the scale’s use in populations or settings not represented in the sample. Over-inclusiveness and self-report bias could result in inflated prevalence estimates or misclassification of risk, which may affect the effectiveness of targeted prevention strategies. Future studies should aim to incorporate qualitative or mixed-methods approaches to help clarify the nuances of exposure and improve item specificity. In addition, triangulating self-report data with administrative records or observational data could help mitigate bias and enhance the accuracy of exposure measurement. The current dataset also could not test true predictive validity of this scale.

In addition, because the scale captures exposure rather than intent or behavior, it should not be used in isolation to make decisions about individuals’ roles in prevention programming. Misinterpretation of high exposure scores as indicators of suitability for intervention could lead to inappropriate or unsafe applications without additional screening.

### Next Steps

Several next steps for validation of the Gun-X Scale are suggested. First, analyses should determine whether the current validity results are seen among participants of different developmental stages, including adolescents and older adults. For example, do the patterns between types of exposure seen in the current study vary by developmental stages? We need to strategically oversample known high-risk populations, such as youth in foster care, juvenile justice settings, and homeless shelters. The Gun-X Scale should also be tested across different settings, such as schools, health clinics, community centers, to see if setting-specific cutoff scores are needed. Practical metrics such as time to complete, acceptability ratings from both youth and providers, and what training scale administrators actually need should also be assessed. Testing whether an ultra-brief 3–4-item version, which captures the most discriminative and representative aspects of bystander exposure, maintains predictive power could be invaluable for busy clinical settings.

We need to use longitudinal data to test temporal stability, to examine whether initial exposure predicts increased risk for gun violence over time (mapping risk trajectories) and to potentially identify critical periods where intervention might be most effective. Network analysis could reveal whether different types of gun violence exposure (witnessing vs. direct threat vs. community violence) create different risk pathways. Finally, research should examine how these exposures relate to subsequent experiences of gun violence victimization and perpetration, including whether Gun-X scores function as markers of environmental risk, pathways to involvement, or opportunities for prevention. Longitudinal designs will be critical for identifying whether and under what conditions exposure predicts divergent outcomes, including risk escalation, resilience, or prosocial intervention.

### Implications for Prevention

First, people, adolescents and adults alike, are aware of a wide range of types of situations involving actual gun violence, inappropriate access and carrying, and risky behaviors. This scale offers a way to conduct landscape assessments of gun violence exposure in communities and thus improve data for (1) prevention actions including awareness building, (2) skill building to improve the effectiveness and safety of bystander intervention to reduce risky gun use by enabling research on how bystander helping unfolds and with what consequences, (3) how that might vary by exposure type, and (4) improving prevention evaluation with better measures of how participation in prevention programs may affect bystander opportunity and exposure. For example, exposures occur in both physical and digital settings suggesting the need to expand prevention messaging to include online environments, such as social media platforms. The Gun-X Scale can be used as a tool to introduce conversations about the safety of the bystander in different scenarios as well as to identify appropriate approaches to help-seeking and intervention.

The importance of having measures that capture a range of bystander opportunities is illustrated in reflections on trends in the past decades toward “lateral surveillance” such as critiques and analyses of the “see something, say something” Department of Homeland Security campaign, or relying on neighbors to look out for one another in ways that may create a backlash of suspicion as well as potential false positives - seeing risky gun use where it does not exist (Andrejevic, 2004; Reeves, 2012). On the other hand, there are discussions about the positive effects of “safety voices” in organizations and research suggesting that forms of interpersonal violence may be reduced by increasing the skills of potential bystanders (Bush et al., 2021; Tedone et al., 2025). The Gun-X Scale is a tool that can support better research on how to train bystanders to helpfully and accurately identify risk and skills to safely intervene and that also itself can be a springboard for community prevention conversations about what situations represent risk or not.

To guide safe and appropriate application in prevention contexts, we propose a three-pathway framework for interpreting Gun-X scores. First, a bystander-training pathway may be appropriate for individuals with moderate levels of exposure who do not report recent involvement in violence and demonstrate willingness to intervene. Second, a support or trauma-informed pathway may be more appropriate for individuals with high levels of exposure accompanied by indicators of distress or burden, reflecting the potential impact of repeated or severe exposure. Third, a risk-monitoring or targeted-prevention pathway may be warranted for individuals with high exposure who also exhibit behavioral risk indicators (e.g., prior gun carrying or other risky behaviors). This framework underscores that exposure alone is insufficient for determining appropriate intervention strategies and highlights the importance of combining Gun-X scores with additional assessment to guide prevention efforts safely and effectively.

## Conclusions

This study offers a promising scale for assessing a broad range of exposure to situations involving gun violence, risky behavior, and inappropriate access or usage. As the gun violence problem continues to evolve, this scale offers a valuable resource for researchers, clinicians, and communities to screen for gun violence awareness and better understand and address how to prevent violence before it occurs. This study provides new evidence that bystander exposure to gun violence is a unified, measurable construct that can be reliably assessed across diverse contexts. This first stage of validation of the Gun-X Scale shows promise for a practical tool that can enable more precise identification of risk and inform the development of targeted, data-driven prevention strategies.

## Data Availability

Individual participant data that underlie the results reported in this article, after de-identification, will be available December 31, 2026 to investigators whose proposed use of the data has been approved by the project leads, who have IRB approval, and who have been approved by an independent review committee identified for this purpose. Proposals should be sent directly to either Bruce Taylor (taylor-bruce@norc.org) or Kimberly Mitchell (kimberly.mitchell@unh.edu) for approval and to gain access. Data requesters will also need to sign a data use agreement. Twelve months after project completion, the data will be available in the Open Science Framework (https://osf.io/) but without investigator support other than deposited metadata.
